# Supplementary material for: Genomic properties of variably methylated retrotransposons in mouse
Source: Mob DNA. 2021 Feb 21;12:6. doi: 10.1186/s13100-021-00235-1 (PMC7898769; doi:10.1186/s13100-021-00235-1)
Supplement: Supplementary file 1 — Additional file 1: CTCF ChIP-seq metadata and genomic features. [file 13100_2021_235_MOESM1_ESM.pdf]

ADDITIONAL FILE 1

	CTCF ChIP reads (#)	CTCF ChIP mapped reads (#)	CTCF ChIP mapped reads (%)	Input reads (#)	Input mapped reads (#)	Input mapped reads (%)	Peaks (# of narrowPeak)
Individual 1	39,064,678	34,767,956	89.0	23,664,646	21,229,047	89.7	50,672
Individual 2	53,600,684	47,478,849	88.6	47,143,046	41,767,434	88.6	59,367
Individual 3	32,241,538	28,780,499	89.3	26,619,852	23,847,345	89.6	47,150
Individual 4	51,457,925	45,475,944	88.4	31,810,231	28,454,053	89.4	44,357
Individual 5	44,457,625	39,480,440	88.8	25,340,000	22,731,001	89.7	39,787
Individual 6	46,121,507	41,529,071	90.0	30,176,868	27,088,768	89.8	51,221
Individual 7	49,823,878	45,184,326	90.7	25,015,109	22,439,766	89.7	55,029
Individual 8	54,730,468	47,628,529	87.0	26,909,635	24,039,485	89.3	41,580

CTCF ChIP-seq metadata:
Read counts and mapping efficiency for eight individual CTCF ChIP-seq and Input libraries, with the count of narrowPeaks from macs2.

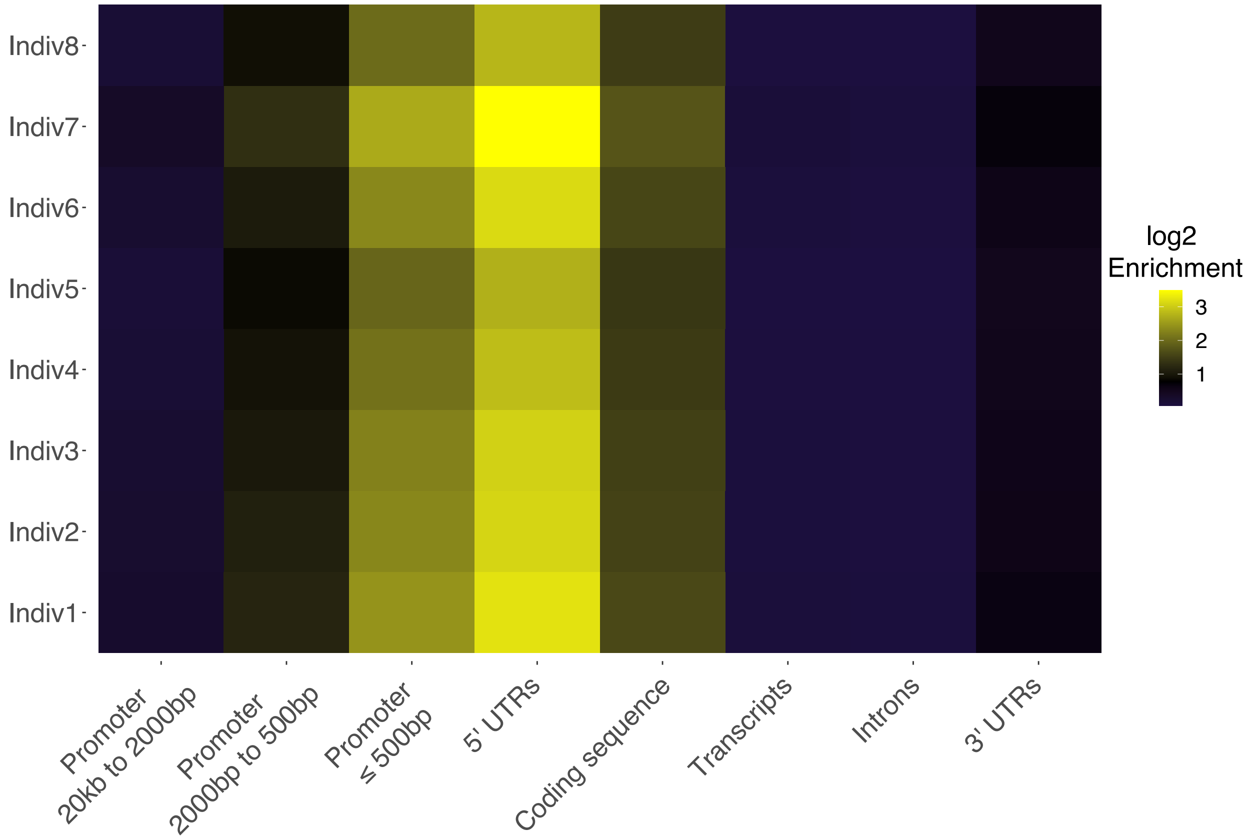

CTCF enrichment in genomic features:
Generated from the “plotRegi” function of the R package ChIPQC v. 1.21.0 using mapped bam files of the eight individual ChIP-seq and Input datasets.
