# Supplementary material for: Genomic properties of variably methylated retrotransposons in mouse
Source: Mob DNA. 2021 Feb 21;12:6. doi: 10.1186/s13100-021-00235-1 (PMC7898769; doi:10.1186/s13100-021-00235-1)

Supplemental Data
Constitutive VM-IAPs (cVM-IAPs)

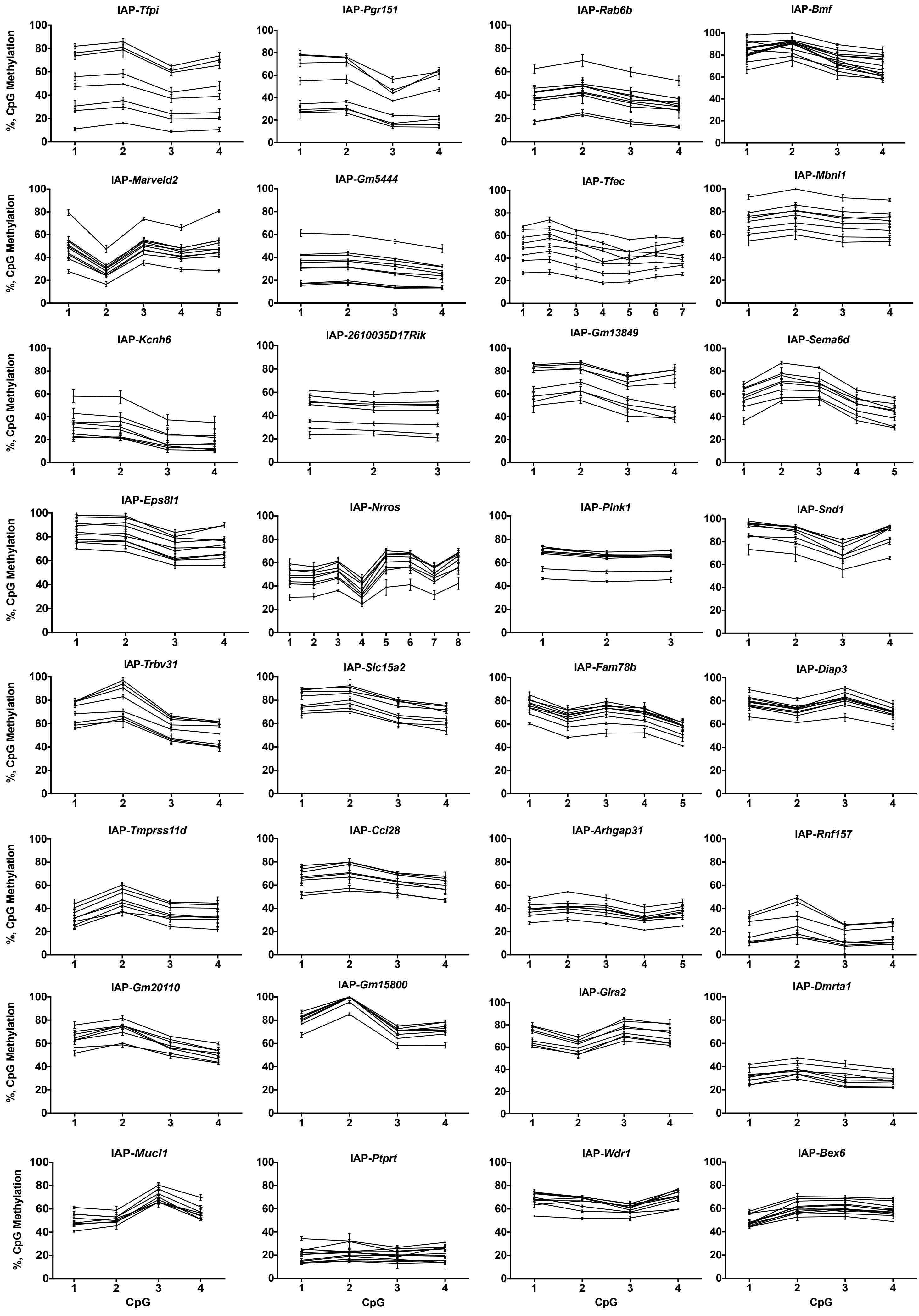

Constitutive VM-IAPs (cVM-IAPs)

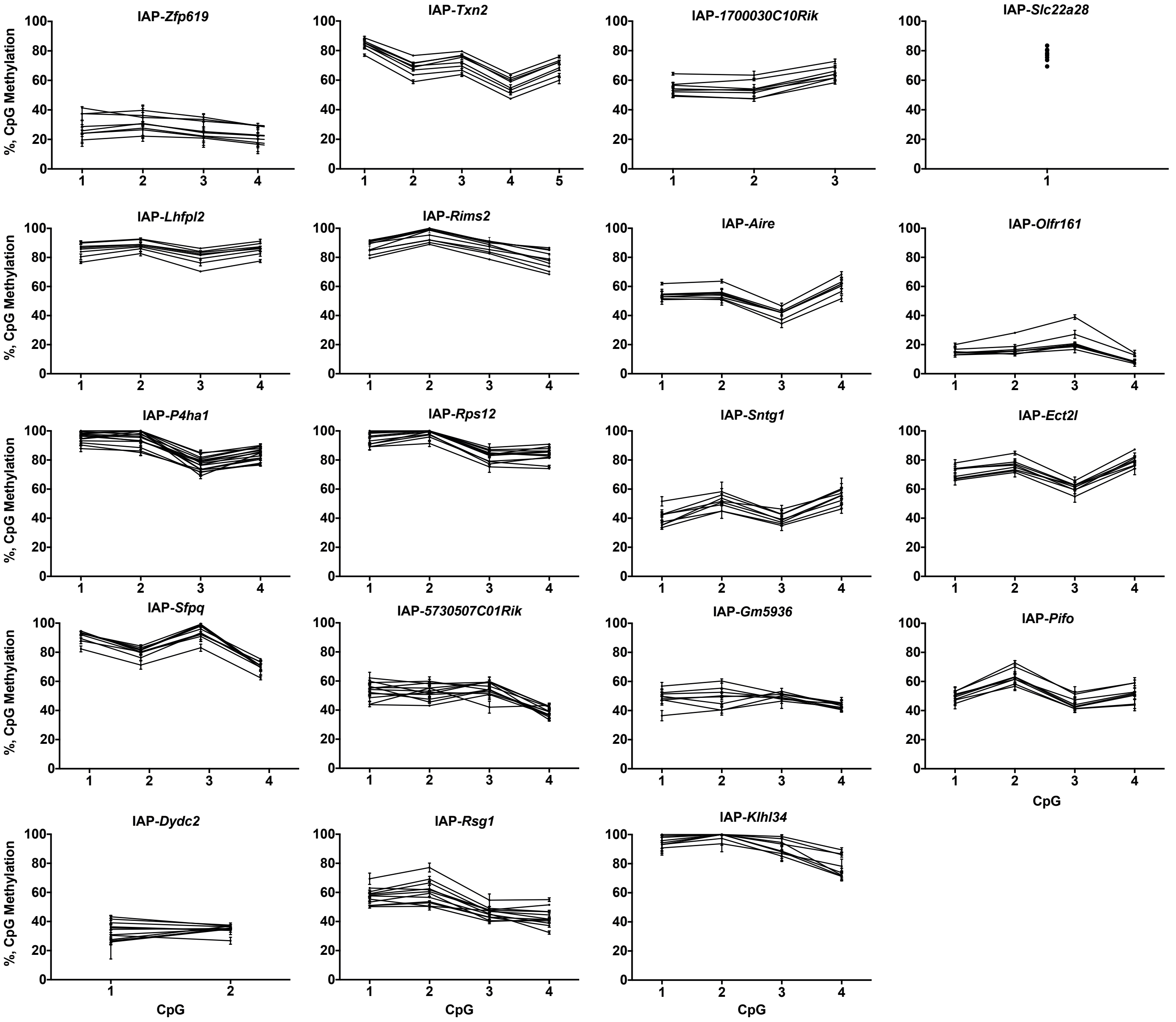

Tissue-specific VM-IAPs (tsVM-IAPs)

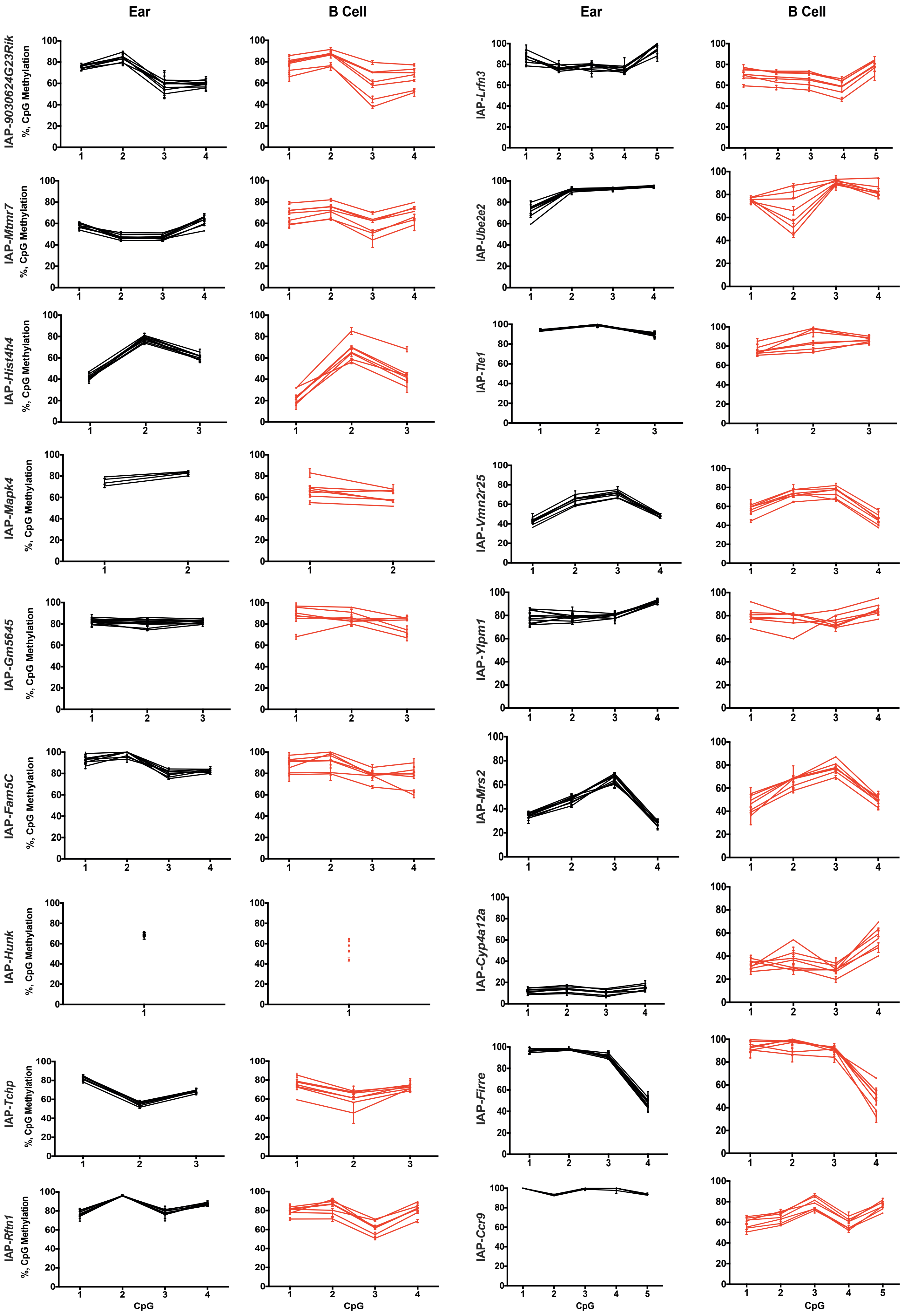

Tissue-specific VM-IAPs (tsVM-IAPs)

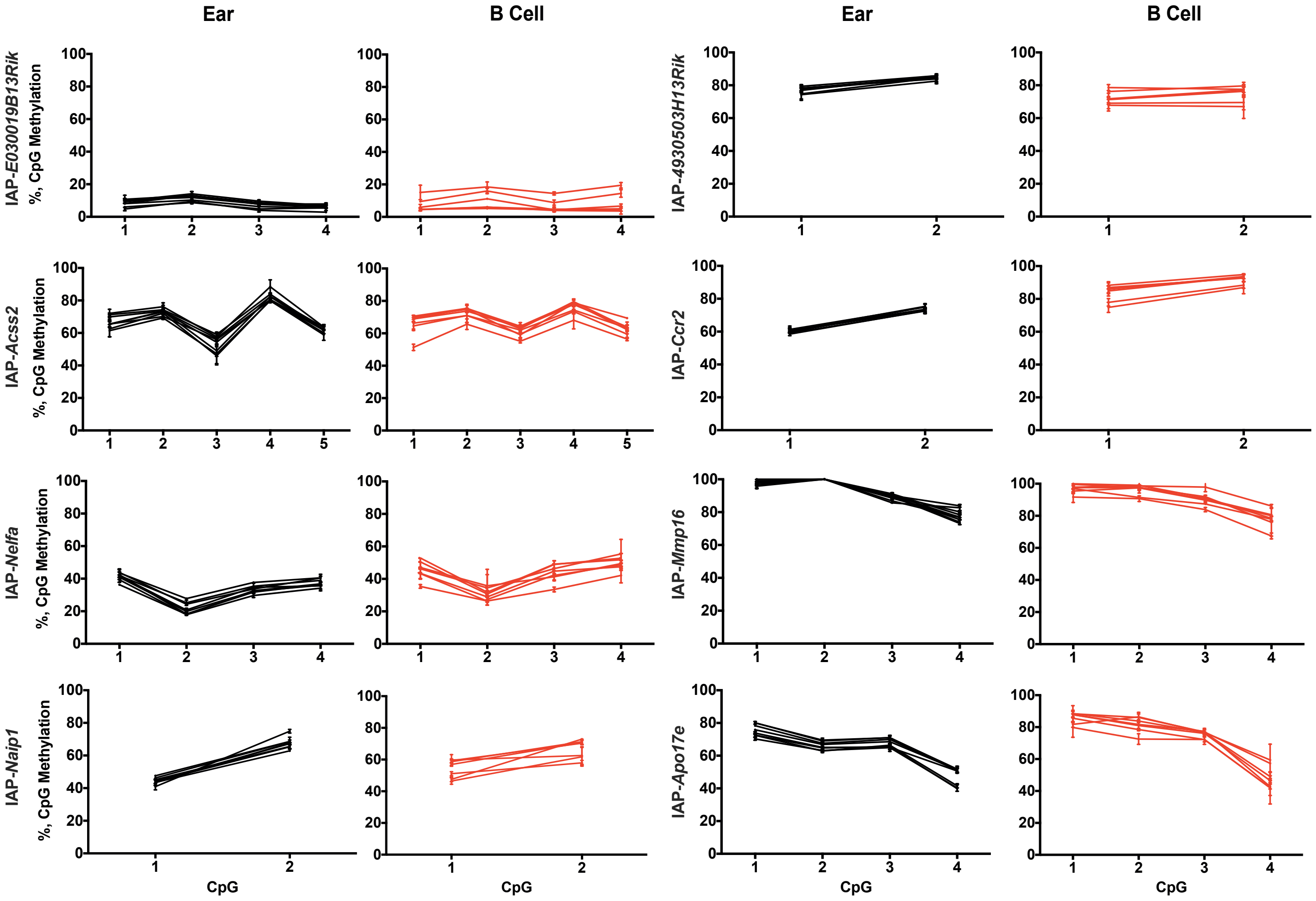

False positive IAP elements

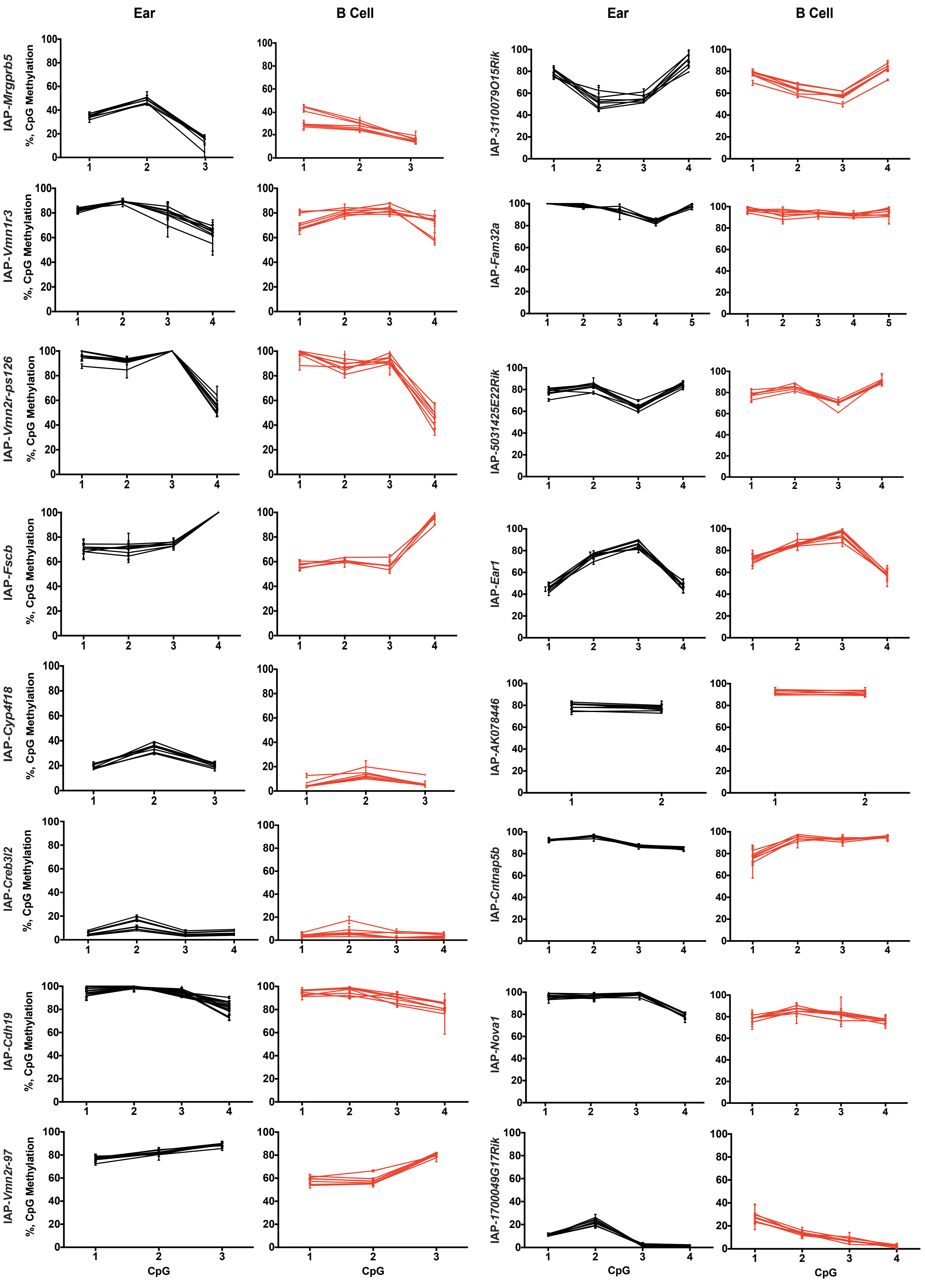

False positive IAP elements

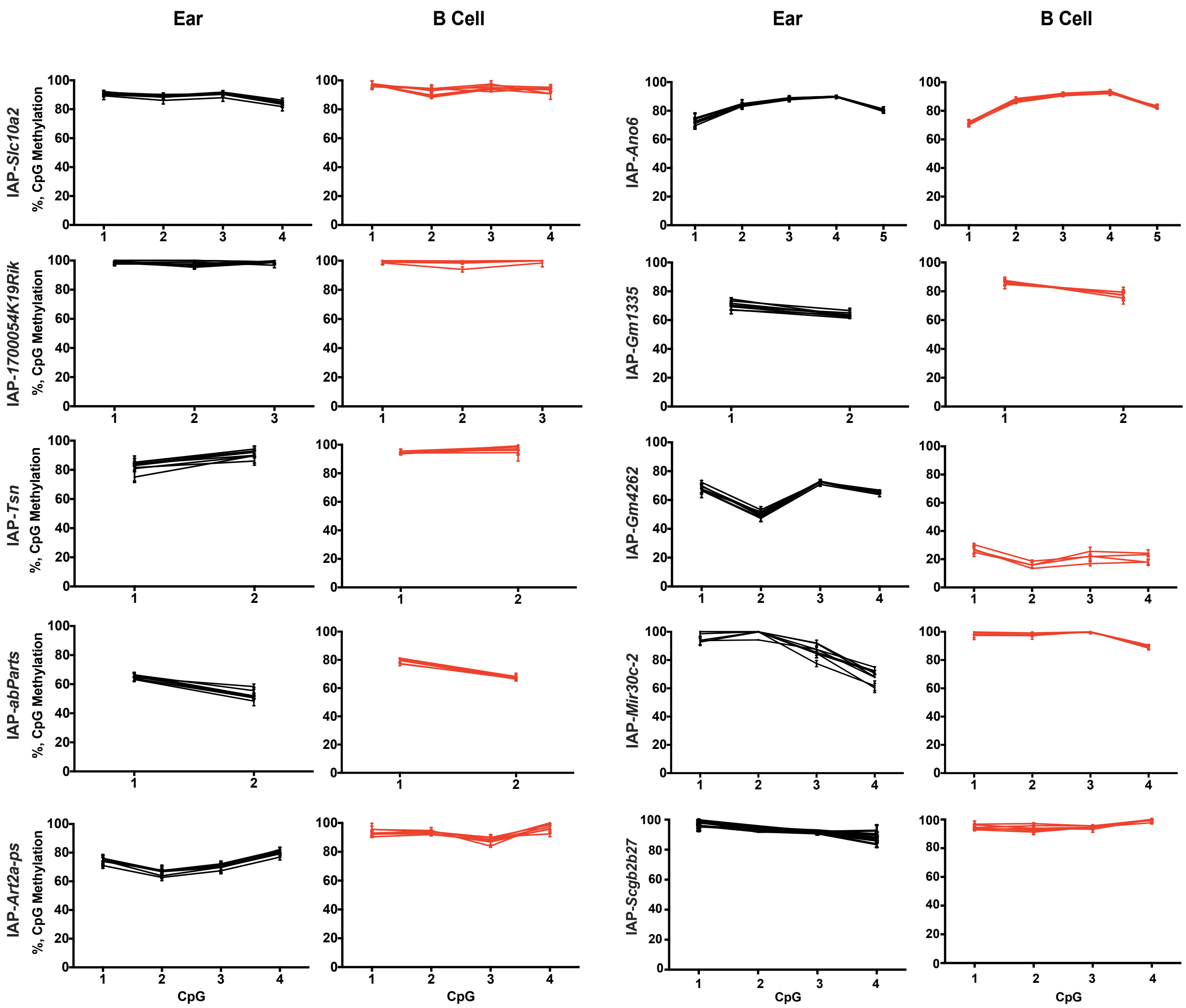

False positive IAP elements - T cells

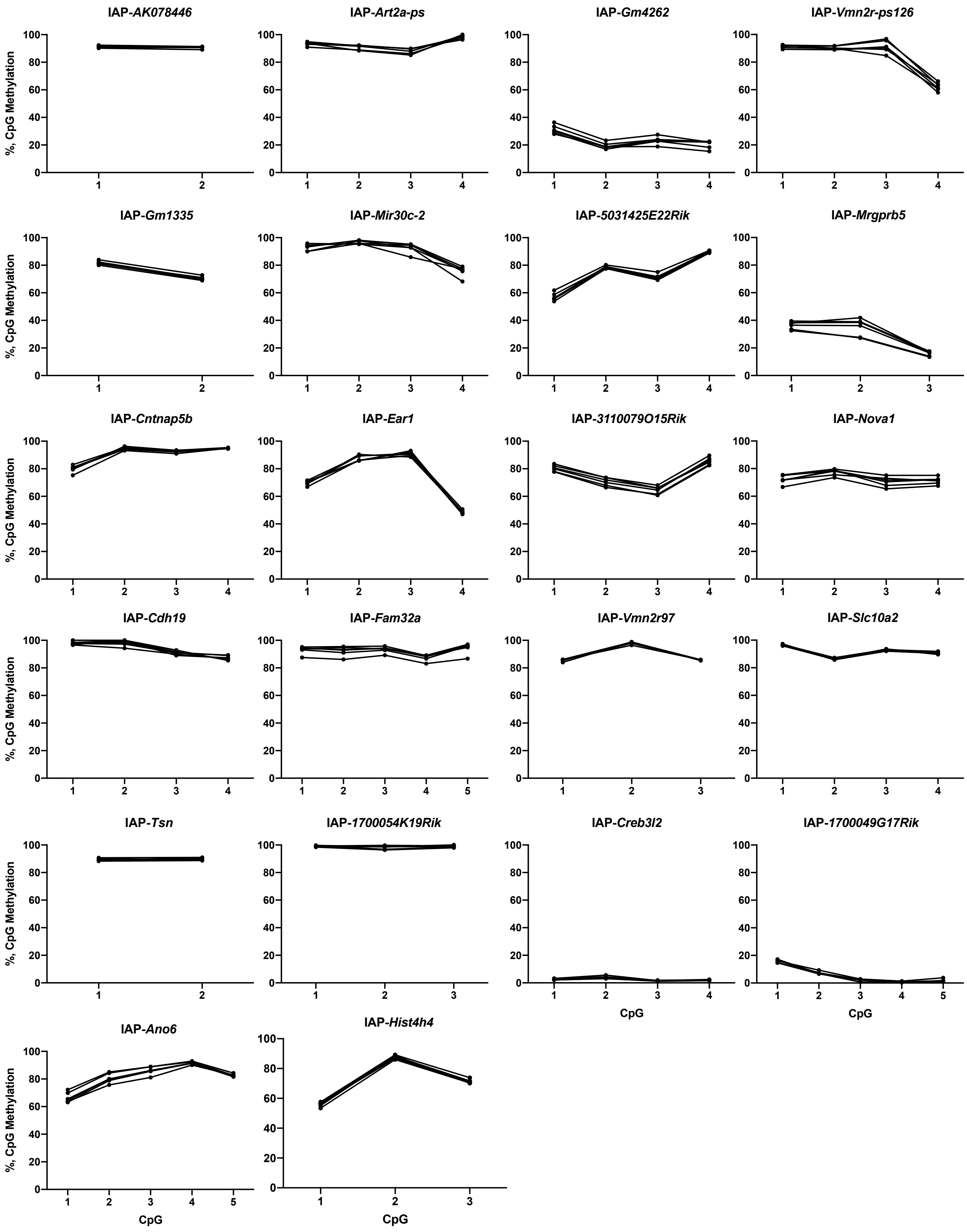

Supplement: Supplementary file 7 — Additional file 7: Supplemental Data. [file 13100_2021_235_MOESM7_ESM.pdf]
